# CT-Guided Iodine-125 Seed Implantation Combined with Anlotinib for Recurrent Retroperitoneal Undifferentiated Pleomorphic Sarcoma: A Case Report

**DOI:** 10.1159/cro/abzag025

**Published:** 2026-08-20

**Authors:** Li Chen, Sheng Lin

**Affiliations:** a Department of Oncology, The Affiliated Hospital of Southwest Medical University, Luzhou, China; b Department of Oncology, Wusheng County People’s Hospital, Guang’an, China

**Keywords:** Undifferentiated pleomorphic sarcoma, Retroperitoneal tumor, Iodine-125 seed implantation, Brachytherapy, Anlotinib

## Abstract

**Introduction:**

Undifferentiated sarcoma is a rare and highly aggressive malignancy with a high risk of postoperative recurrence and limited treatment options. The management of recurrent retroperitoneal sarcoma remains challenging. CT-guided iodine-125 (I-125) seed implantation provides localized brachytherapy, while anlotinib, a multi-target tyrosine kinase inhibitor, has demonstrated efficacy in soft-tissue sarcomas. However, evidence regarding their combined use is limited.

**Case Presentation:**

We report a patient with recurrent retroperitoneal undifferentiated sarcoma following prior surgical resection. The recurrence was identified on follow-up imaging as a retroperitoneal mass with irregular margins and heterogeneous enhancement, and was deemed unresectable due to its proximity to critical structures. After multidisciplinary evaluation, the patient underwent CT-guided I-125 radioactive seed implantation for local tumor control. The procedure was successfully completed without complications. Subsequently, the patient received oral anlotinib therapy. The combined treatment was well tolerated, with no severe adverse events observed. Serial follow-up imaging demonstrated a reduction in tumor size and sustained local disease control, indicating a favorable therapeutic response.

**Conclusion:**

This case suggests that CT-guided I-125 seed implantation combined with anlotinib may be a safe and effective therapeutic option for patients with recurrent retroperitoneal undifferentiated sarcoma who are not candidates for surgery. Further studies are warranted to confirm these findings.

## Introduction

Undifferentiated pleomorphic sarcoma (UPS) is an aggressive subtype of soft-tissue sarcoma, accounting for approximately 10–20% of adult soft-tissue sarcomas [1]. Retroperitoneal involvement is rare and often diagnosed at an advanced stage because of the large potential space and nonspecific early symptoms [2]. Complete surgical resection with negative margins remains the cornerstone of treatment; however, local recurrence is common and is associated with poor prognosis. Management of recurrent retroperitoneal UPS is challenging, particularly in elderly patients who are not suitable for repeat surgery or high-dose external-beam radiotherapy [3–5]. CT-guided iodine-125 (I-125) seed implantation is a form of brachytherapy that allows continuous low-dose irradiation with relative sparing of adjacent organs [6, 7]. Here, we report a rare case of recurrent retroperitoneal UPS successfully managed with CT-guided I-125 seed implantation combined with anlotinib, achieving durable disease stabilization.

## Case Report

A 71-year-old man was admitted in February 2024 for evaluation of colonic polyps. He had no specific abdominal symptoms, including pain, weight loss, or gastrointestinal obstruction. His past medical history was unremarkable. Physical examination revealed no palpable abdominal mass or tenderness.

A routine preoperative abdominal computed tomography (CT) scan incidentally demonstrated a well-defined soft-tissue mass measuring approximately 73 × 64 mm located anterior to the left kidney, with heterogeneous enhancement and compression of adjacent structures (Fig. 1). No distant metastases were identified.

**Fig. 1.**
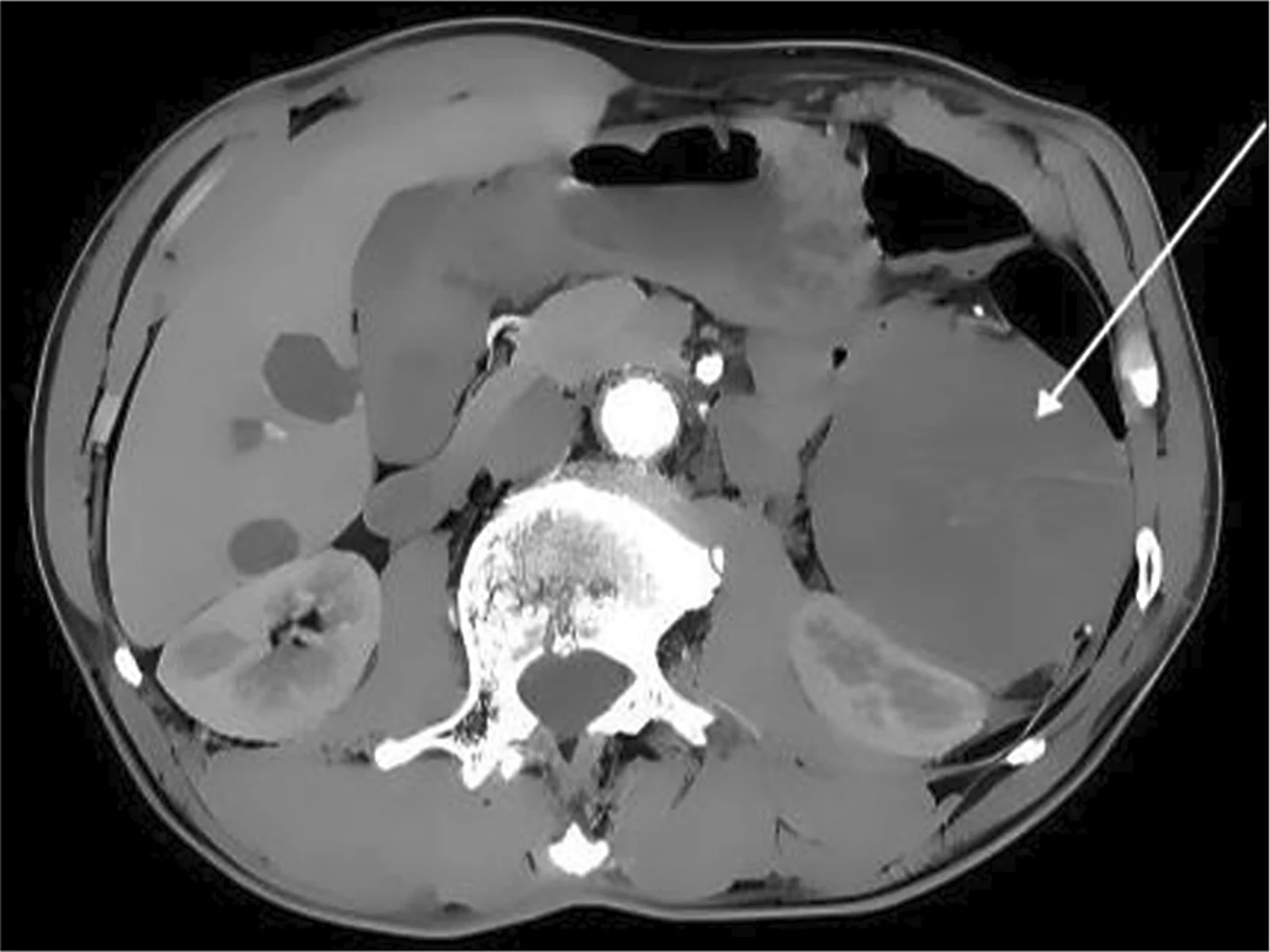
Contrast-enhanced abdominal CT showing a heterogeneous soft tissue mass anterior to the left kidney at initial presentation. The arrow indicates the tumor.

Based on imaging findings, the differential diagnosis included retroperitoneal soft-tissue sarcoma, lymphoma, gastrointestinal stromal tumor, and metastatic disease. Surgical excision was performed to establish a definitive diagnosis.

In March 2024, the patient underwent retroperitoneal tumor resection with ileostomy and adhesiolysis. Gross pathological examination revealed a solid mass measuring approximately 10 × 7.5 × 7 cm with a gray-white to gray-yellow cut surface. Histopathological evaluation showed a pleomorphic spindle cell malignant tumor. Immunohistochemical staining demonstrated CD68 (−/+), desmin (−), SMA (−/+), S-100 (−), HMB45 (−), DOG1 (−), EMA (−), Ki-67 approximately 40% positive, and p53 approximately 20% weak to moderate positivity. These findings were consistent with a diagnosis of UPS of the retroperitoneum.

Postoperatively, the patient received two cycles of adjuvant chemotherapy with doxorubicin (50 mg on day 1) and cyclophosphamide (0.7 g on day 1) every 21 days. Chemotherapy was discontinued due to patient preference.

Follow-up CT imaging in June 2024 revealed a recurrent heterogeneous mass adjacent to the pancreatic tail, measuring approximately 51 × 26 mm, consistent with local recurrence (Fig. 2). Multidisciplinary evaluation concluded that repeat surgical resection was not feasible.

**Fig. 2.**
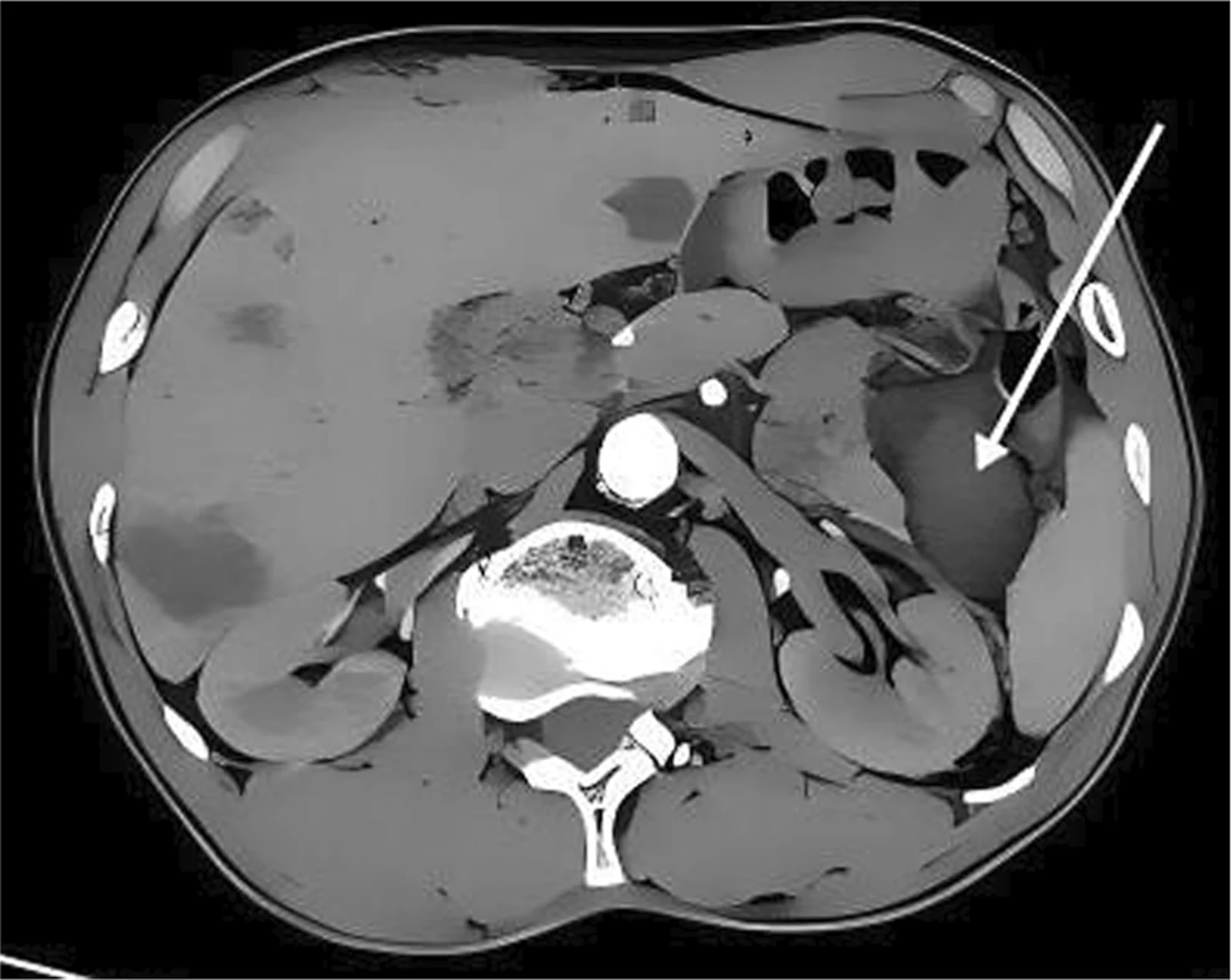
Follow-up CT demonstrating recurrent tumor adjacent to the pancreatic tail. The arrow indicates the recurrent lesion.

In July 2024, the patient underwent CT-guided I-125 seed implantation. The sealed titanium seeds (Tianjin Saide Biopharmaceutical Co., Ltd., China) contained I-125 loaded on a silver core; each seed had an apparent activity of 0.8 mCi (29.6 MBq), dimensions of 4.50 × 0.80 mm, a principal photon energy of 35.5 keV, and a physical half-life of 59.6 days. Preoperative contrast-enhanced CT images (1.5-mm slice thickness) were imported into a treatment planning system (HGGR-2000; Zhuhai Hokai Medical Instruments Co., Ltd., China). The prescribed dose was 100 Gy, with planning objectives of D90 ≥ 100 Gy and V100 ≥ 90%. The calculated plan required 30 seeds (total activity, 888 MBq). Under local anesthesia and real-time CT guidance, an 18-G needle was advanced percutaneously to the margin of the left retroperitoneal lesion. Seeds were deployed from deep to superficial while withdrawing the needle, with inter-seed spacing of 0.5–1.5 cm to achieve a homogeneous dose distribution and limit cold and hot spots. Immediate post-implantation CT was used to assess seed distribution, migration, hemorrhage, and adjacent-organ injury. The images were reimported into the planning system for post-implant dosimetric verification, confirming that D90 and V100 met the planned objectives. Standard radiation-protection measures were used throughout the procedure, and the patient received radiation-safety counseling. Oral anlotinib was subsequently initiated at 12 mg once daily.

The patient tolerated the I-125 seed implantation procedure well, with no significant perioperative complications or treatment-related pain. One month after implantation, CT imaging showed gas absorption within the lesion and multiple high-density foci corresponding to implanted seeds (Fig. 3). Subsequent follow-up CT scans at 4, 7, 10, and 12 months demonstrated a gradual reduction in viable tumor components, with the lesion largely replaced by fatty density and stable residual changes measuring approximately 46 × 32 mm (Fig. 4).

**Fig. 3.**
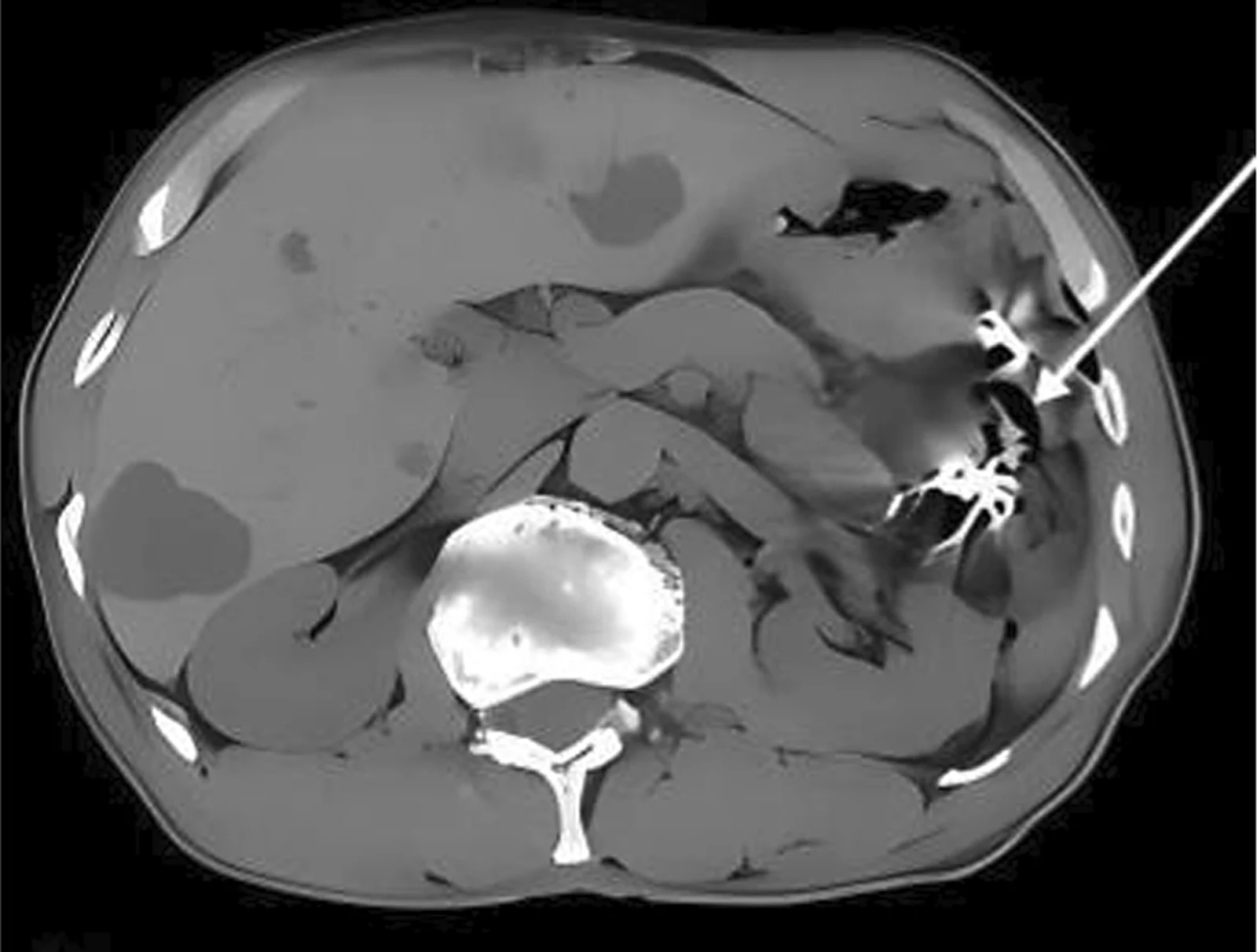
CT image obtained 1 month after iodine-125 seed implantation showing intratumoral high-density seeds and treatment-related changes. The arrow indicates the implanted seeds.

**Fig. 4.**
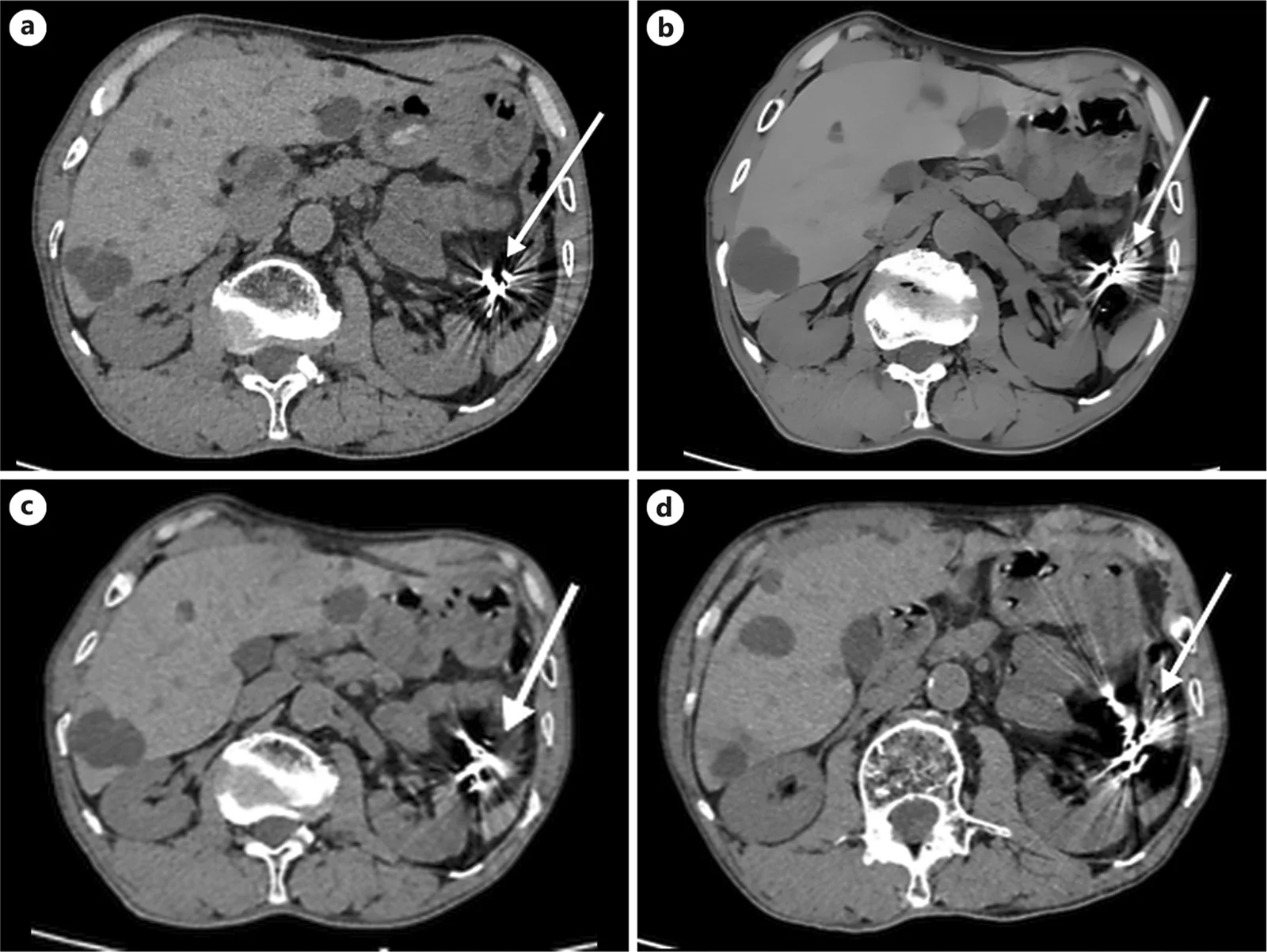
Serial follow-up CT scans at 4 (**a**), 7 (**b**), 10 (**c**), and 12 (**d**) months demonstrating stable disease with fatty replacement and no evidence of progression. Arrows indicate the residual lesion.

Patient-reported pain was assessed with the Verbal Rating Scale (VRS): pain improved from grade II before treatment (moderate pain requiring analgesics and disturbing sleep) to grade 0 after treatment. No grade 3 or 4 adverse events were observed according to CTCAE v5.0. The patient reported only mild fatigue and grade 1 hand-foot skin reaction, which were managed symptomatically without dose reduction or treatment interruption. No radiation enteritis, myelosuppression, or other serious complications occurred. At the last follow-up, the patient remained clinically stable without disease progression.

Clinical timeline: The patient underwent retroperitoneal tumor resection in March 2024, and histopathology confirmed UPS. Two cycles of adjuvant chemotherapy were completed, with the last cycle administered in May 2024. Local recurrence (approximately 51 × 26 mm) was detected in June 2024 and was considered unresectable. CT-guided I-125 seed implantation (30 seeds; prescribed dose, 100 Gy) was performed in July 2024, followed by oral anlotinib. During serial follow-up through July 2025, the recurrent lesion decreased in size and remained stable (approximately 46 × 32 mm), without metastasis; the best response was stable disease. We completed the CARE checklist to ensure all relevant information for this case report was included and it is available as online supplementary file (for all online suppl. material, see https://doi.org/10.1159/cro/abzag025).

## Discussion

Retroperitoneal UPS is an uncommon and highly aggressive malignancy characterized by a high rate of local recurrence, even after macroscopically complete surgical resection [8, 9]. Owing to the large potential space of the retroperitoneum, tumors often reach a considerable size before diagnosis, and local recurrence remains the predominant cause of treatment failure and mortality [10]. Current international guidelines emphasize complete surgical excision with negative margins as the cornerstone of management; however, evidence supporting adjuvant radiotherapy or chemotherapy in this setting remains limited and controversial, particularly because of toxicity concerns related to surrounding critical organs [11, 12].

Management of locally recurrent retroperitoneal UPS is especially challenging when repeat surgery is not feasible. External-beam radiotherapy may provide local control in selected cases, but adequate dosing is often restricted by radiosensitivity of adjacent organs, such as the kidney, bowel, and pancreas [13–15]. Other local treatment modalities, including radiofrequency ablation or transarterial therapies, have shown limited efficacy due to uncertain tumor response and the relatively poor vascularity of retroperitoneal sarcomas [16–18]. Consequently, there is a clear need for alternative local treatment strategies that can achieve tumor control while minimizing treatment-related morbidity.

For high-risk or metastatic disease, systemic chemotherapy may be considered, although its benefit in UPS is not uniformly established. Doxorubicin plus ifosfamide with mesna (AIM), a standard option for adult soft-tissue sarcoma, can increase response rates but is associated with substantial hematologic and nonhematologic toxicity [19–23]. In this patient, recurrence occurred shortly after doxorubicin-based adjuvant chemotherapy, and the expected benefit of rechallenge was uncertain. Molecularly targeted treatment is also difficult because UPS typically exhibits complex, heterogeneous genomic alterations without a consistent actionable driver [24]. Immunotherapy may benefit selected patients, but responses are inconsistent and depend on features of the immune microenvironment; many sarcomas exhibit limited immune infiltration or immunosuppressive features [25–27]. Other local options include external-beam radiotherapy, thermal ablation, and embolization, but their feasibility is constrained by proximity to the kidney, bowel, and pancreas, lesion geometry, and tumor vascularity. These considerations supported a combined local brachytherapy and systemic antiangiogenic strategy in this case.

CT-guided I-125 seed implantation represents a form of brachytherapy that delivers continuous low-dose-rate irradiation with a limited radiation radius, allowing precise dose deposition within the tumor while sparing surrounding normal tissues [28–30]. Previous studies have demonstrated the feasibility and safety of I-125 brachytherapy in unresectable or recurrent retroperitoneal tumors, but reports focusing specifically on UPS remain scarce. In the present case, CT-guided I-125 seed implantation was selected after multidisciplinary discussion and was well tolerated, achieving sustained local disease stabilization without significant complications.

In addition to local therapy, systemic treatment remains important for recurrent UPS. Anlotinib was selected because it inhibits multiple kinases involved in tumor angiogenesis, proliferation, and the tumor microenvironment, including VEGFR1–3, FGFR1–4, PDGFRα/β, c-Kit, RET, and DDR1 [31, 32]. Preclinical data indicate potent inhibition of VEGFR2-, PDGFRβ-, and FGFR1-mediated angiogenesis, with greater antiangiogenic activity than several comparator tyrosine kinase inhibitors at equivalent concentrations [33]. Antitumor activity has also been demonstrated in osteosarcoma and synovial sarcoma models through VEGFR2/MET and GINS1-related pathways [34, 35]. Clinically, anlotinib has shown manageable toxicity and disease-control activity in unresectable or metastatic soft-tissue sarcoma, including as maintenance therapy and in well-differentiated/dedifferentiated liposarcoma [36–39]. Given the patient’s age, early recurrence after doxorubicin-based chemotherapy, reluctance to continue cytotoxic therapy, and need for a tolerable outpatient regimen, anlotinib was favored over further chemotherapy. The combination with brachytherapy was intended to control local tumor burden while suppressing angiogenesis and potential systemic disease.

This report has several limitations. As a single observational case without a concurrent control, it cannot distinguish the individual contributions of seed implantation and anlotinib or establish causality. Follow-up was limited to 12 months; longer observation is required to assess durable local control, delayed radiation injury, progression-free survival, and overall survival. UPS lacks a uniform driver alteration, and molecular profiling and tumor mutational burden were not assessed; future studies should incorporate high-throughput sequencing and dynamic biomarkers. The findings therefore should not be generalized to all patients with UPS or other soft-tissue sarcomas. Nevertheless, the tolerability and sustained local control observed here provide a hypothesis-generating rationale for prospective real-world studies or retrospective cohorts in carefully selected older or frail patients with unresectable locally recurrent retroperitoneal sarcoma. Such studies should define molecularly informed selection criteria, optimal sequencing, and the relative contribution of each treatment component.

## Acknowledgments

The authors would like to express gratitude to all individuals and institutions that contributed to the completion of this paper. Their support, guidance, and encouragement throughout the research process are deeply appreciated.

## Statement of Ethics

This study was performed in accordance with the Declaration of Helsinki. This study was approved by the Ethics Committee of Wusheng County People’s Hospital (Ethics No.: 20251120008). Written informed consent was obtained from the patient who is the subject of this study for publication of this case report.

## Conflict of Interest Statement

The views expressed in this report are solely those of the authors and do not represent the official positions of any affiliated institutions.

## Funding Sources

No funding was received.

## Author Contributions

Li Chen: conceptualization, data curation, investigation, methodology, writing – original draft, and writing – review and editing. Sheng Lin: conceptualization, data curation, investigation, writing – original draft, and writing – review and editing.

## Data Availability

The data that support the findings of this report are not publicly available to maintain the privacy of the patient. They are available from the corresponding author upon reasonable request.
